# Nitrogen Application Can Optimize Form of Selenium in Soil in Selenium-Rich Areas to Affect Selenium Absorption and Accumulation in Black Wheat

**DOI:** 10.3390/plants12244160

**Published:** 2023-12-14

**Authors:** Weilin Kong, Ruiwen Huo, Yu Lu, Zhenjie Fan, Runqing Yue, Aixia Ren, Linghong Li, Pengcheng Ding, Yongkang Ren, Zhiqiang Gao, Min Sun

**Affiliations:** 1 College of Agriculture, Shanxi Agriculture University, Taigu, Jinzhong 030801, China; 2 Collaborative Innovation Center for High-Quality and Efficient Production of Characteristic Crops on the Loess Plateau Jointly Built by Provinces and Ministries, Taigu, Jinzhong 030801, China; 3 Key Laboratory of Functional Agriculture of Ministry of Agriculture and Rural Affairs, Taigu, Jinzhong 030801, China; 4 Yangquan Agricultural Technical Service Center, Yangquan 045000, China

**Keywords:** black wheat, nitrogen and selenium interaction, path analysis, selenium-rich soil, soil selenium form

## Abstract

The composition and form of selenium in the soil have significant effects on the selenium content of crops. In this study, we investigated the selenium absorption pathway in plants by studying the interaction between nitrogen fertilizer and soil selenium. Our results showed that the selenium concentration enrichment factors (CEF) varied within the same region due to nitrogen fertilizer application, where they ranged from 1.33 to 5.02. The soil selenium flow coefficient (mobility factor, MF) increased with higher nitrogen application rates. The sum of the MF values for each soil layer treated with nitrogen application rates of 192 kg hm^−2^ and 240 kg hm^−2^ was 0.70, which was 64% higher than that for the control group with no nitrogen application. In the 0–20 cm soil layer, the highest summed water-soluble and exchangeable selenium and relative percentage of total selenium (12.45%) was observed at a nitrogen application rate of 240 kg hm^−2^. In the 20–40 cm soil layer, the highest relative percentage content of water-soluble and exchangeable selenium and total selenium (12.66%) was observed at a nitrogen application rate of 192 kg hm^−2^. Experimental treatment of black wheat with various concentrations of sodium selenite showed that selenium treatment at 50 μmol L^−1^ significantly increased the reduced glutathione (GSH) levels in the leaves and roots of seedlings, where the GSH contents increased by 155.4% in the leaves and by 91.5% in the roots. Further analysis of the soil–black wheat system showed that nitrogen application in selenium-rich areas affected the soil selenium flow coefficient and morphological composition, thereby changing the enrichment coefficient for leaves (0.823), transport capacity from leaves to grains (–0.530), and enrichment coefficient for roots (0.38). These changes ultimately affected the selenium concentration in the grains of black wheat.

## 1. Introduction

Selenium (Se) is a vital element for plant growth and development, as well as an essential trace element for human and animal life activities [1]. An inadequate intake of selenium can increase the risk of hyperglycemia and cardiovascular disease, and selenium deficiency is also associated with Keshan disease [2]. Approximately one billion people worldwide consume insufficient selenium in their diets [3,4], and the negative effects of selenium deficiency on health have been increasing in recent decades. Some regions in China, New Zealand, and Europe are characterized by low dietary selenium levels [5]. In China, around 51% of the soil is deficient in selenium, and 39–61% of residents consume insufficient selenium. Humans and animals primarily obtain selenium from food and drinking water. The main cause of human selenium deficiency is the lack of selenium in foods such as cereals [6], which is closely related to the form of selenium in the soil. In addition to the total selenium content of the soil, the form of selenium is considered the most important factor that affects its availability, migration, and transformation [7]. Thus, the total soil selenium alone does not represent its bioavailability and bioavailable selenium is a key parameter that determines the mobility of selenium in the soil and its absorption by plants.

Wheat (*Triticum aestivum* L.) is considered an important source of selenium supplementation for humans [8]. However, the selenium content of Chinese wheat is currently low and insufficient to meet the health needs of the population [9]. Biofortified selenium-enriched foods can help alleviate selenium deficiency [10] but they also pose serious ecological challenges. The soil is the primary source of selenium for crops, and thus the selenium content of soil significantly determines the selenium levels in agricultural products and overall biological health. Natural selenium-rich areas have unique advantages for cultivating pure natural, green, and pollution-free selenium-rich crops. Therefore, utilizing natural selenium-rich soil to enhance the selenium content of the edible plant parts is the most cost-effective, efficient, and safe approach for increasing selenium levels in plants. Black wheat is a particularly nutritious variety that is rich in proteins, amino acids, dietary fiber, minerals, and vitamins [11]. The grains of black wheat contain abundant natural pigments, mainly anthocyanins, as well as high levels of micronutrients, thereby making it a valuable source for selenium supplementation [12]. Consequently, it is important to investigate the characteristics of soil selenium in selenium-enriched areas and understand the transport process in black wheat.

Nitrogen fertilizer is essential for plant growth and it is widely applied in agricultural production. However, in China, there are increasing concerns regarding excessive nitrogen fertilizer inputs and the high labor cost of fertilization despite the steady growth in wheat yields. The average nitrogen application rate for wheat in the North China Plain regions is 424 kg hm^−2^, which is much higher than the nutrient requirement. In addition, it is important to note that the supply of nitrogen increases the availability of nitrogen-containing substances for crops but also affects the absorption of other mineral elements. In particular, Fallahi et al. [13] showed that nitrogen fertilizer can stimulate vegetative growth, thereby leading to increased competition for mineral elements between vegetative and storage organs, and hindering the distribution of mineral elements to storage organs. Excessive nitrogen fertilizer application can also reduce the iron and manganese contents of apple fruit [13]. Soltanpour et al. [14] demonstrated that N application could increase the wheat yield but it had no significant effect on the selenium content of grain. By contrast, Govasmark et al. [15,16] showed that increasing the amount of nitrogen fertilizer applied could significantly enhance the selenium content of wheat grains. In addition, the application of nitrogen fertilizer to wheat in the jointing stage can increase the protein contents of the stems and leaves, thereby providing more sites for selenium absorption to ultimately promote the transfer of selenium to the grain, and this process reduces the selenium concentrations in the stems and leaves. The inconsistent conclusions obtained in previous studies regarding the effects of nitrogen fertilizer application on the absorption of mineral elements crops necessitate further research into how nitrogen application affects the absorption, transport, and distribution of selenium in wheat.

In the present study, we aimed to promote the healthy growth of high-quality wheat by focusing on a natural selenium-rich area and conducting tests using the black wheat variety Yunhei 14207. Different nitrogen fertilizer concentrations were applied before obtaining mature black wheat samples and soil samples from the 0–100 cm soil layer. We also assessed environmental indicators for the selenium-rich soil to understand their effects on the migration and transformation of selenium. Moreover, a comprehensive path analysis was conducted to examine the transport and distribution of selenium in the soil–wheat system. We studied the changes in the selenium contents of black wheat grains under the interaction between nitrogen fertilizer and selenium, and the associated mechanism was investigated in experiments using black wheat seedlings cultured with different concentrations of sodium selenite. The results obtained in this study may provide technical support for natural and healthy selenium biofortification in black wheat.

## 2. Results

### 2.1. Changes in Environmental Characteristics in 0–100 cm Soil Layer of Selenium-Rich Soil under Different Nitrogen Application Rates

The basic chemical properties of the soil samples are shown in Table 1. The soil samples had weak alkaline pH values (pH range: 7.02–7.6). The pH increased with the soil depth under N192, N216, and N240, but the overall differences were not significant. The available phosphorus content of the soil decreased as the soil depth increased, particularly in the 40–60 cm layer where it was 46.7–68.1% lower compared with the layer above. Compared with N0, the available phosphorus contents were higher under the other nitrogen fertilizer treatments, but there were no significant differences among the different nitrogen application levels. The highest available soil phosphorus content was found in the topsoil (0–20 cm) under N168, with a value of 67.23 mg kg^−1^. The total selenium content of the soil decreased as the soil depth increased, thereby indicating its accumulation in the upper layer. The mean total selenium contents in each soil layer were 14.15%, 15.21%, 19.40%, and 37.25% lower, respectively, compared with the upper soil layer, and increasing the nitrogen fertilizer application rate increased the accumulation of selenium in the upper layer.

**Figure 1:**
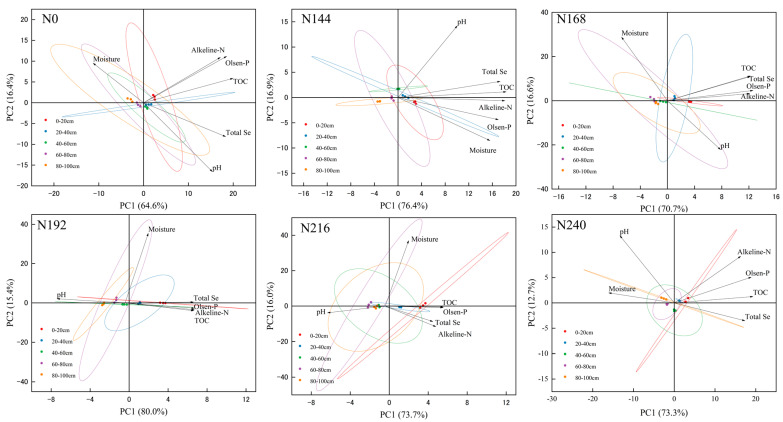
Principal component analysis based on effects of different nitrogen treatments on characteristics of soil samples.

Significant correlations were found between the total selenium contents in the soil samples and environmental factors (Figure 1). Principal component analysis detected negative correlations between soil moisture and the total selenium contents under N0, N168, and N240. The pH value was negatively correlated with the soil selenium contents under N192, N216, and N240. In addition, the available phosphorus, alkali-hydrolyzed nitrogen, and organic carbon contents were significantly positively correlated with the selenium contents under different nitrogen application rates.

### 2.2. Differences in Selenium Concentration Enrichment Factor (CEF) Values in 0–100 cm Layer of Selenium-Rich Soil under Different Nitrogen Application Rates

**Figure 2:**
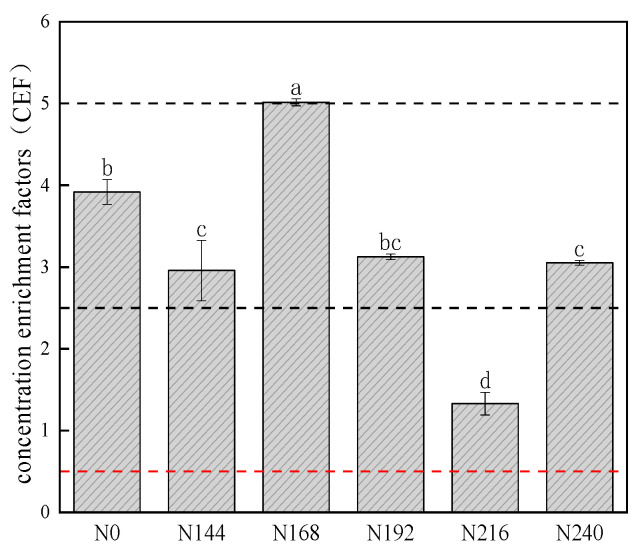
CEF values for Se vs. Co(C) under different nitrogen application rates. Values represent mean ± standard error (*n* = 3). Different letters indicate significant differences (*p* < 0.05) between treatments. The red dashed line indicates that CEF is equal to 0.5; black dashed line indicates that CEF is equal to 2.5.

The majority of CEF values for Se range from 0.5 to 2.5. This suggests that there is a correlation between the Se content in the topsoil and the Se content in the subsoil. However, when the CEF value falls below 0.5, it signifies that the Se content in the topsoil is lost compared to the deep soil. Values of CEF > 2.5 indicate that nitrogen positively impacts the enrichment of selenium in the topsoil. Values of CEF > 5 indicate that selenium exceeds the standard, and there is a possibility of high pollution [17]. Figure 2 shows that the application of nitrogen fertilizer in the same region led to variations in the selenium CEFs. The CEF had a value of 5.02, whereas the CEF values for the other treatments were below 5, thereby suggesting that selenium was highly enriched in the surface soil but there was not a significant pollution risk.

### 2.3. Differences in Soil Selenium Mobility (MF) and Forms of Selenium under Different Nitrogen Application Rates

**Figure 3:**
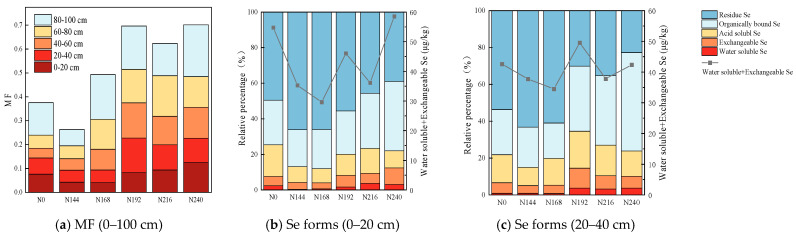
Analysis of changes in MF (**a**) and selenium forms in soil (**b**,**c**). The left *y*-axis represents the relative proportions of selenium, i.e., the percentage of each selenium form among the total selenium. The lines represent the changes in the sum of the water-soluble selenium and exchangeable selenium in soil under different nitrogen application rates, as shown on the right *y*-axis.

The MF values for the 0–100 cm soil layers under different nitrogen application rates are shown in Figure 3a. The MF value increased as the nitrogen application rate increased. The sum of the MF values under N192 and N240 was 0.70, which was 64% higher than that under N0. The second highest MF was obtained under N216, with a value of 0.62, which was also 64% higher than that under N0. The two most important soil layers for wheat growth and nutrient absorption are the 0–20 cm soil plow layer and 20–40 cm sub-tillage layer. The effects of different nitrogen application rates on the forms of selenium and the sum of the water-soluble and exchangeable selenium in the topsoil (b) and sub-soil (c) samples are shown in Figure 3. The water-soluble Se in soil and sediment mainly comprises SeO_4_^2−^, with some SeO_3_^2−^ and soluble organic Se. The results showed that in the 0–20 cm soil layer, the percentage of water-soluble selenium relative to total selenium was highest under N216 at 3.68%. In the 20–40 cm soil layer, the percentage of water-soluble selenium relative to total selenium was highest under N240 (3.33%), but there was no significant difference compared with N192 (3.21%). As the nitrogen application rate increased, the change in the graph plotting the sum of the water-soluble and exchangeable selenium had a “W”-type shape. In the 0–20 cm soil layer, the sum of the water-soluble and exchangeable selenium and the relative percentage of total selenium was highest under N240 (12.45%), followed by N216 and N192. In the 20–40 cm soil layer, the percentage of water-soluble and exchangeable selenium relative to total selenium was highest under N192 (12.66%), followed by N216 and N240. The sum of Se in the acid soluble state, organic bound state, and residual state under each nitrogen fertilizer treatment in the soil plow layer (except under N240) and sub-tillage layer (except under N192) accounted for more than 90% of the total forms, thereby indicating that most of the Se was fixed by the soil and not readily absorbed by plants. Thus, it is necessary to change the form of Se through human activities and the effects of environmental factors in order to promote its absorption by plants.

### 2.4. Changes in Selenium Absorption and Transport in Black Wheat Plants under Different Nitrogen Application Rates

**Figure 4:**
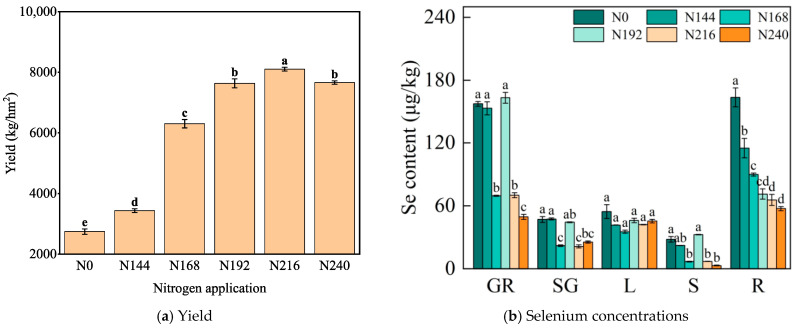
Changes in Yield under different nitrogen application rates (**a**). Changes in selenium concentrations in different wheat organs under different nitrogen application rates (**b**). GR denotes grain, SG denotes glume, L denotes leaf, S denotes stem, and R denotes root. Different letters indicate significant differences (*p* < 0.05).

The yield was highest under N216 at 8045.32 kg hm^−2^. The second is N240, but not significantly different from under N192 (Figure 4a). The selenium concentration in the grain was greatest under N192 compared with the other nitrogen application rates. The selenium concentration in the grain was highest under N192 at 169.10 μg kg^−1^, but not significantly different from those under N0 and N144. However, the selenium concentration in the roots decreased as the nitrogen application rate increased. The selenium concentration in the roots was lowest under N240 at 57.31 μg kg^−1^, but not significantly different from those under N216 and N192. In Yunhei 14207 plants, the highest selenium concentrations were found in the grains and roots, followed by the leaves and glumes, and the lowest concentration in the stems. The nitrogen application rate had no significant effect on the selenium concentration in the leaves, but the selenium concentration in the roots decreased as the nitrogen application rate increased (Figure 4b).

The transport factor (TF) and bioenrichment factor (BCF) values for selenium (Se) in wheat plants under different nitrogen application rates are presented in Table 2. Clear decreases in BCF were found in each organ under N168 and N240. Pearson’s correlation coefficients were calculated to examine the relationships between the selenium concentrations in the grain and selenium TFs. The results indicated that the selenium concentration in the grain had negative correlations with the stem to grain TF (r = −0.83, *p* ≤ 0.001), stem to rachis glume TF (r = −0.73, *p* ≤ 0.001), and stem to leaf TF (r = −0.8, *p* ≤ 0.001), but positive correlations with the leaf to grain TF (r = 0.91, *p* ≤ 0.001), glume to grain TF (r = 0.62, *p* ≤ 0.01), leaf to rachis glume TF (r = 0.86, *p* ≤ 0.01), and root to stem TF (r = 0.69, *p* ≤ 0.001). Stepwise regression was conducted to determine the relationships between the selenium concentration in the grain and Se TFs. The stepwise regression analysis with the selenium concentration in the grain as the dependent variable and seven TFs as independent variables showed that the TFs from leaves to grains had significant effects on the selenium concentration in the grain. The following equation was derived from the analysis: Y_grain_= −1.143 + 44.802 × TF_leaf-grain_ (R^2^ = 0.874). The TF_leaf-grain_ values under N144 and N192 were 3.68 and 3.54, respectively. The enrichment coefficient for selenium also reflected the level of difficulty for selenium migration in the selenium–black wheat system to some extent. As the nitrogen application rate increased, BCF_root_ decreased, with the highest value under N0 (0.226) and the lowest values under N240 and N168 (0.122).

### 2.5. Comprehensive Path Analysis Based on Soil Selenium Contents, Nitrogen Application Rate, and Black Wheat Responses

**Figure 5:**
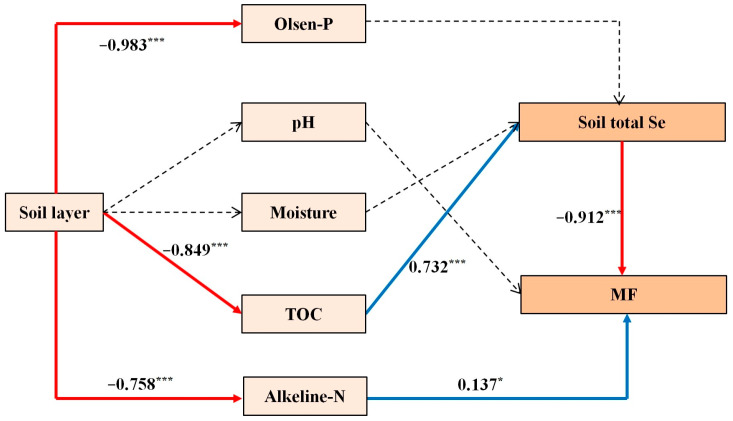
Path analysis based on effects of soil environmental factors on soil selenium in the soil layer. A blue line indicates a positive flow direction and a red line indicates a negative flow direction. The solid line represents a significant correlation, the dashed line represents a possible flow direction, but there is no correlation between the data. * *p* < 0.05; *** *p* < 0.001.

Path analysis was conducted to further investigate the effects of the soil layer and related environmental indicators on the soil selenium contents in the selenium-enriched area by examining the relationships between the soil layer, environmental factors, and soil selenium content (path represented by a solid line in Figure 5). The model’s fit, discriminant validity, and reliability were assessed using fitting and internal consistency statistics. The goodness of fit was 0.887 (considered acceptable within the range of 0.5–0.8), while the normed fit index was 0.945 (greater than 0.8), the Tucker–Lewis index was 0.932 (greater than 0.8), and the comparative fit index was 0.959 (greater than 0.8). All of these indicators satisfied the standard criteria for an acceptable level of model fitting. Thus, in this study, the effect of the soil layer on soil selenium in selenium-rich areas could be attributed to two main pathways. The first pathway involved the impact of the soil layer on the total selenium content, which subsequently affected the form of selenium (MF). The second pathway involved the impact of the soil layer on alkali-hydrolyzed nitrogen, which then affected the form of selenium (MF).

**Figure 6:**
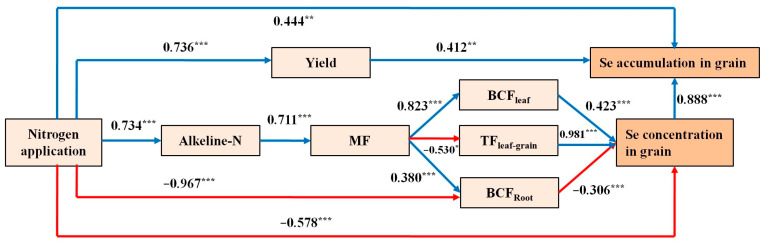
Integrated path analysis for topsoil and wheat. A blue line indicates a positive flow direction and the red line indicates a negative flow direction.* *p* < 0.05; ** *p* < 0.01; *** *p* < 0.001.

After nitrogen application, the regulation of selenium accumulation in black wheat grains was influenced by both the whole plant and the soil (Figure 6). The model’s fit, discriminant validity, and reliability were evaluated using fitting and internal consistency statistics. The goodness of fit was 0.787 (acceptable range: 0.5–0.8), while the normed fit index was 0.88 (greater than 0.8), the Tucker–Lewis index was 0.959 (greater than 0.8), and the comparative fit index was 0.976 (greater than 0.8). All of these indicators satisfied the standard criteria for an acceptable level of model fitting. The impact of nitrogen application on selenium accumulation in the grain was primarily influenced through two pathways. First, nitrogen application affected both the yield (0.736) and selenium accumulation in the grain (0.412). Second, the amount of nitrogen applied influenced the alkali-hydrolyzable nitrogen content (0.734), which then affected the form of selenium (MF) (0.711). These effects resulted in changes in the enrichment coefficient for leaves (0.823), transport capacity from leaves to grains (−0.530), and enrichment coefficient for roots (0.38). Consequently, the changes in the selenium concentration in the grain ultimately led to changes in selenium accumulation (0.888). The total effect of the nitrogen application rate on selenium accumulation in the grain was 0.3631.

### 2.6. Effects of Sodium Selenite Solution on Glutathione (GSH), Total Antioxidant Capacity (T-AOC), and Hydrogen Peroxide (H_2_O_2_) Contents of Black Wheat Seedlings

**Figure 7:**
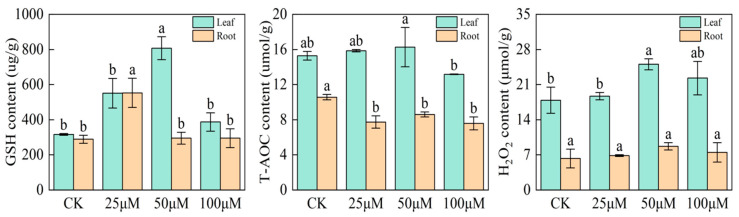
Effects of sodium selenite treatment on GSH, T-AOC, and H_2_O_2_ contents in roots and leaves of wheat seedlings.Here, 25 μM denotes 25 μmol L^−1^, 50 μM denotes 50 μmol L^−1^, and 100 μM denotes 100 μmol L^−1^. Different lowercase letters represent different organs significantly different (*p* < 0.05).

Compared with the control group, selenium treatment under 25 μmol L^−1^ Se, 50 μmol L^−1^ Se, and 100 μmol L^−1^ Se increased the GSH contents by 74.2%, 155.4%, and 22.5%, respectively. In particular, the GSH content of the leaves increased significantly under 50 μmolL^−1^ selenium. Selenium treatment also increased the GSH contents in the roots of black wheat seedlings compared with the control, where 50 μmol L^−1^ selenium increased the GSH content of the roots by 91.5%. Selenium treatment significantly reduced the T-AOC contents of black wheat seedlings by 26.8%, 18.63%, and 28.24% under 25 μmol L^−1^ Se, 50 μmol L^−1^ Se, and 100 μmol L^−1^ Se, respectively, compared with the control. The T-AOC content decreased significantly in the leaves under 100 μmol L^−1^ selenium, where the T-AOC content decreased by 13.8% compared with the control. The H_2_O_2_ content of the leaves increased by 40.1% with 50 μmol L^−1^ selenium treatment, but there were no significant differences in the H_2_O_2_ contents in the roots of black wheat seedlings. These results indicate that sodium selenite could improve the antioxidant capacity of black wheat leaves to some extent (≤50 μmol L^−1^) but without significantly reducing the antioxidant capacity of the roots (Figure 7). 

## 3. Discussion

### 3.1. Effects of Environmental Factors on Soil Total Selenium Content and MF in Vertical Distribution of Soil

Selenium is subject to constant adsorption and desorption processes in the soil [18]. The redox reactions associated with these processes are influenced by the soil acidity and alkalinity [19], which then affect the bioavailability of soil selenium. Wang et al. [20] showed that the adsorption of selenium by soil decreased as the pH value increased. Therefore, it is considered that the bioavailability of selenium is higher in neutral or alkaline environments compared with acidic environments. In the present study, there was no significant variation in the pH value within the same area and ecological environment, thereby indicating that differences in the bioavailability of selenium were not significant. However, the soil pH in the study area was above 7, which enhances the bioavailability of selenium in selenium-rich areas. Studies have demonstrated that selenium in the soil is readily adsorbed by organic matter and converted into an organic bound state, thereby reducing the mobility of selenium in the soil and decreasing its absorption by plants [21,22]. In the present study, the total organic carbon content had a significant effect on the soil selenium content, with an effect size of 0.732.

A study conducted in the Mancos region of southwestern USA showed that the soil selenium content varied as the depth increased, and these variations were attributed to differences in leaching caused by rainfall [23]. Similarly, Gabos et al. [24] found that the selenium content of oxidized soil in Brazil increased with the depth, and the changes were correlated with the soil cation exchange capacity, pH value, and organic matter. However, we obtained contrasting results in the present study, with negative impacts of the soil layer depth on the total selenium and MF value in soil. The soil layers significantly influenced the total organic carbon and alkali-hydrolyzed nitrogen contents, which then affected the total selenium and MF values. The effects of phosphorus, pH, and water on soil selenium were found to be insignificant. It should be noted that the influence of phosphorus on soil selenium may be more prominent in the plow layer. Moreover, variations in the pH and water contents in different geographical environments can lead to substantial differences in the soil selenium contents. In the present study, the samples were collected from the same ecological environment in the same region, thereby minimizing the significance of differences in the pH and water content.

### 3.2. Effects of Nitrogen Application on Form of Soil Selenium

After continuous leaching, the forms of Se in Se-rich soil were categorized into five fractions comprising the water-soluble fraction, exchangeable fraction, carbonate and iron-manganese oxide bound fraction, organic matter bound fraction, and residual fraction. The quantity of each form of Se was correlated with the total Se content. Among the various forms of Se, water-soluble Se is the most readily absorbed and utilized by plants, and thus it represents the available Se. By contrast, the residual Se (including sulfide Se) is the least readily absorbed by plants. The exchangeable Se can be utilized by plants, but its usage is restricted under specific conditions. Acid-soluble and organic Se are forms of Se released under strong acid and strong oxidizing conditions, respectively, and they are generally not absorbed by plants, but they can be converted into forms that are absorbed by plants under certain conditions. Therefore, the MF is better for representing the effectiveness of selenium utilization, where a higher MF indicates a greater possibility of selenium absorption by plants. Comparison of the MF values for selenium under the six nitrogen fertilizer treatments showed that medium nitrogen (N192) and high nitrogen (N216 and N240) application rates were more beneficial for activating soil selenium. Under the conditions in the present study, increasing the MF value through an appropriate nitrogen fertilizer input amount, specifically by increasing the proportion of water-soluble and exchangeable forms among the total selenium, was an effective agronomic measure for activating soil selenium, and thus the absorption of selenium by black wheat was enhanced.

### 3.3. Effects of Nitrogen Application on Selenium Translocation and Distribution in Organs of Black Wheat

Plant roots absorb selenate in the soil through active energy consumption [25], selenite through passive diffusion [26], and organic selenium through amino acid transporters in the cell membrane [27]. Exogenous selenium sprayed as fertilizer is absorbed by mesophyll cells through diffusion, and these cells can absorb inorganic, organic, and even nano-sized selenium [28,29,30,31]. Leaves also play a crucial role in selenium transport from selenium-rich soil. Selenium is directly transported from the leaves to the grains through the phloem, or via phloem during grain filling and ripening to adjacent nodes, and then transported upward [32]. In the present study, the migration coefficient from leaves to grains directly affected the selenium content of the grain. Based on the results obtained by regression analysis, we conclude that the transport of selenium from the leaves to grains had the strongest association with the selenium concentration in wheat grains, with a positive correlation. However, the differences in the selenium concentrations were not significant in the leaves, and thus leaves may primarily facilitate transformation of the form of selenium and its conduction. In addition, as the nitrogen fertilizer application rate increased, the enrichment coefficient for wheat roots tended to decrease, thereby indicating the difficulty of selenium migration in the selenium–black wheat system. The BCF_root_ value was smaller under N192 (0.128) but the BCF_grain_ value was the highest (0.292), and thus N192 may have reduced the storage of selenium in the roots as well as increasing root absorption and transport, which led to greater selenium transportation and storage in the grains.

### 3.4. Effects of Nitrogen Application on Selenium Accumulation in Surface Soil and Grain

Compared with fixed elements, CEF values can be better for indicating the enrichment and dilution of topsoil in relation to the geochemical background. These values are influenced by atmospheric, biological, and human activities [33]. In previous studies, CEF values were mainly used to assess the impacts of human activities on heavy metal pollution in topsoil [17,34,35]. Bottom soil represents the composition of the lithosphere and the geological background, and it is affected less by supergene processes and human activities [36]. In this study, Co was used as the reference element and CEF values were utilized to reflect the changes in Se in the surface soil due to human activities, such as nitrogen fertilizer application. Yu et al. [37] found that Se tended to accumulate in the upper soil layer in Mianyang, Sichuan, and attributed this phenomenon to human activities and bioaccumulation. Hao et al. [38] found that the selenium content was high in the surface soil in Langao County, which may have been associated with stone, coal, and human activities. Lei et al. [39] detected the highest selenium content in the surface soil in Aksu in Xinjiang and attributed this to agricultural activities. In the present study, we mostly obtained Se CEF values ranging from 2.5 to 5 with no agricultural measures apart from nitrogen application. Our results showed that nitrogen fertilizer had a positive impact on the enrichment of selenium in the surface soil. Comparisons of the CEF values for selenium under the six different nitrogen fertilizer treatments showed that low nitrogen (N0/N144) and medium nitrogen (N168/N192) application rates were more effective in enriching total selenium in the surface soil. In the soil–plant system, nitrogen application mainly influenced the black wheat yield and soil selenium flow coefficient (MF), which then affected the ability of the leaves and roots to accumulate and transfer selenium to ultimately influence the accumulation of selenium in the grains.

### 3.5. Effects of Nitrogen Application on Selenium Accumulation in Soil Plow Layer and Grain

Glutathione peroxidase (GSH-Px) is a crucial peroxidase enzyme that is widely distributed in plants. The active center of GSH-Px is selenocysteine and its activity reflects the selenium (Se) levels in plants. Selenium is a component of the GSH-Px enzyme system, which converts GSH into GSSG, and reduces toxic peroxides to non-toxic hydroxyl compounds. This process helps to protect the structure and function of cell membranes from interference and damage by peroxide [40]. GSH-Px is also a regulated storage form of selenium, where GSH-Px can decompose and release selenium when it is required. In the present study, treatment with sodium selenite at less than 50 μmol L^−1^ significantly increased the GSH contents of the leaves and roots of black wheat seedlings by 155.4% and 91.5%, respectively. Selenite is the primary form of selenium in soil and it is readily available to plants. Selenite is commonly found in soil in temperate humid areas, but it tends to bind with clay minerals/iron-aluminum oxides in the soil, which reduces its effectiveness [41]. In the present study, we found that nitrogen application could influence the soil flow coefficient to lead to an increase in the soil water-soluble and exchangeable selenium contents. Consequently, the selenite content available in the soil to plants also increased. Increasing the sodium selenite content enhanced the antioxidant capacity of black wheat leaves and also increased the GSH contents of the leaves and roots. Thus, the increased GSH-Px activity could alleviate selenium poisoning and facilitate the rapid release of selenium to allow more selenium to be transported to the grains.

## 4. Materials and Methods

### 4.1. Experimental Area

The experiment was conducted in Hongtong County, Shanxi Province, China (36°18′ N, 111°38′ E). The Hongtong test area is located in the western part of the Huang-Huai-Hai River Basin, which is classified as a winter wheat production area within the Huang-Huai region. The altitude of the test area is 460 m. The annual frost-free period is 210 days, with an average annual temperature of 12.1 °C and average annual precipitation of 441.5 mm. This region has a warm temperate semi-humid continental monsoon climate. The planting pattern in this area is based on a two-cropping system, where winter wheat is sown in mid-October and harvested during early June in the following year, and maize is sown in mid-to-late June and harvested during early October in the same year. All straw from the crops was crushed and returned to the field. The soil in this area is classified as loam. Prior to the experiment, the soil selenium contents were determined as 582.42 μg kg^−1^ in the 0–20 cm soil depth and 547.75 μg kg^−1^ in the 20–40 cm soil depth, and thus the selenium content was high according to Blazina [42].

### 4.2. Experimental Method

The wheat variety used in this study was Yunhei 14207 (YH14207, black wheat). Yunhei 14207 is a winter variety with a growth period of 245 days. The seedling has a semi-creeping habit and medium tillering tendency. In the later stages, the stems and leaves are non-waxy, with narrow and long flag leaves, a neat panicle layer, and good maturity. The spikes are spindle-shaped with white shells, short awns, and dark blue spikey grains.

Experiment 1: Field Experiment

A single-factor completely randomized design was applied in the experiment. Six nitrogen application levels of 0, 144, 168, 192, 216, and 240 kg hm^−2^ (ratio of base to topdressing = 6:4) were tested (denoted as N0, N144, N168, N192, N216, and N240, respectively). The plot area was 36 m^2^ (4 m × 9 m) and three replicates were performed for each treatment. The experiment was conducted with wide and uniform sowing (row spacing = 25 cm, seedling bandwidth = 8 cm). Before sowing, P_2_O_5_ at 150 kg hm^−2^ and K_2_O at 90 kg hm^−2^ were applied as basal fertilizer. The basic seedling density was 315 × 104 plants hm^−2^. Irrigation with 60 mm of water was applied at the overwintering stage, jointing stage, and flowering stage. Topdressing was conducted using nitrogen fertilizer together with irrigation at the jointing stage. Other field management practices such as weeding and pest control were carried out according to local guidelines. Wheat was sowed on 27 October 2021 and harvested on 15 June 2022.

Experiment 2: Hydroponic Experiment

For this experiment, wheat seeds with the same size were carefully selected and disinfected using 5 mg L^−1^ sodium hypochlorite solution for 5 min. The seeds were then rinsed with deionized water and soaked in deionized water for 24 h. After the wheat seeds absorbed the water, they were placed in a plastic tray with gauze and germinated in an incubator at a temperature of 25 °C. After germination, seedlings with the same size were selected and transferred to a wheat hydroponic box, which was then placed in a well-lit greenhouse. The culture temperature was set at 22 °C, and the plants were exposed to light for 16 h per day. The plants were cultured using 1/2 Hoagland nutrient solution with various concentrations of sodium selenite, and the culture solution was replaced every three days. Harvesting was conducted at the four-leaf stage for the wheat seedlings. Four treatments were tested in this experiment: control (CK), 25 μmol L^−1^ Se, 50 μmol L^−1^ Se, and 100 μmol L^−1^ Se.

### 4.3. Sampling and Chemical Analysis

Sample collection: Twenty plants with uniform growth were selected at the mature stage. These plants were carefully rinsed with deionized water and then dried. Organs and tissues were sampled, including root, stem, leaf, rachis glume, and grain samples. The samples were deactivated at 105 °C and dried to constant weight at 75 °C. A plant powder prototype (miniature plant sample mill; FZ102, Beijing Yongliang Medical Instrument Co., Ltd., Beijing, China) was used to crush the samples to determine their selenium contents.

Plot grain yield was determined by harvesting all plants in an area of 10 m^2^ (located at the center of the plot to eliminate marginal effects). They were shelled using a machine and the grain was air-dried before grain yield determination.

**Figure 8:**
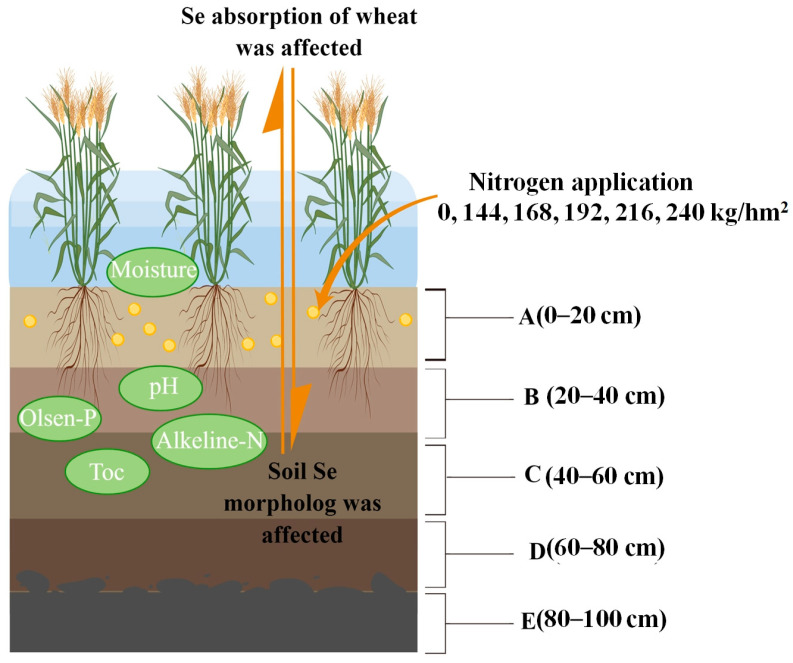
Experimental design.

Vertical soil profiles were collected from the six nitrogen fertilizer application treatments using soil sampling drills. The profiles were divided into five soil layers: 0–20 cm, 20–40 cm, 40–60 cm, 60–80 cm, and 80–100 cm. The soil was naturally dried in the shade, before grinding and sieving to determine soil environmental indicators comprising the moisture content, pH, available phosphorus, alkali-hydrolyzable nitrogen, organic carbon, and forms of soil selenium (Figure 8).

Plant and soil selenium content: To determine the selenium content, 0.3000 g of black wheat or soil sample was digested with HNO_3_. The sample was digested using a microwave digestion instrument (CEM-Mars One, CEM Company, Boston, MA, USA). After cooling, the sample was filtered and digested, and the volume was adjusted to 10 mL with deionized water. The selenium content was determined by hydride generation atomic fluorescence spectrometry according to the Chinese national standard method GB/T35876-2018 [43] (AFS-9780, Haiguang Company, Beijing, China).

Forms of soil selenium: The forms of selenium in soil were determined using the continuous extraction analysis method described by Martens [44]. This method involves extracting selenium in different forms: water-soluble state (H_2_O, 1 h at room temperature), exchangeable state (0.1 mol L^−1^ K_2_HPO_4_–KH_2_PO_4_, 2 h at room temperature), acid-soluble state (3 mol L^−1^ HCL, 50 min at 90 °C), organic bound state (0.1 mol L^−1^ K_2_S_2_O_8_, 2 h at 90 °C), and residual state (HNO_3_ digestion).

The reference conditions used for the atomic fluorescence instrument in this study were as follows: selenium-containing hollow cathode lamp; negative high voltage of 340 V; lamp current of 100 mA; raw temperature of 800 °C; furnace height of 8 mm; carrier gas flow rate of 500 mL min^−1^; and protective gas flow of 1000 mL min^−1^. The selenium concentration was determined using the standard curve method, where the peak area of the reading was used for calculations. The instrument settings comprised a delay time of 1 s, read time of 15 s, and filling time of 8 s. The injection volume was 2 mL.

Water culture experiment: Wheat seedlings at the three-leaf stage were tested in a water culture experiment. The levels of reduced glutathione (GSH), total antioxidant capacity (T-AOC), and hydrogen peroxide (H_2_O_2_) were measured in the leaves and roots.

The GSH content was determined as described by Cui et al. [45]. The frozen tissues of leaves and roots were subjected to homogenization in cold 5% 5-sulfosalicylic acid. After homogenization, the resultant mixture was centrifuged at 20,000× *g* for 15 min at 4 °C. The supernatant obtained from the centrifugation was collected for further analysis of GSH. Total glutathione (GSH+GSSG) was determined in the homogenates spectrophotometrically at 412 nm, using glutathione reductase, 5,5′-dithio-bis-(2-nitrobenzoic acid) (DTNB), and NADPH. The determination of GSSG was accomplished using the same method but in the presence of 2-vinylpyridine. The reduced GSH content was then calculated by subtracting GSSG from the total glutathione amount.

T-AOC was determined using the method described by Pellegrini et al. [46]. The method employed in this study is focused on reducing the complex of Fe^3+^-2,4,6-tripyridyl-s-triazine (TPTZ) to its ferrous state at a low pH. The frozen tissues of leaves and roots were homogenized in water. After homogenization, the resulting mixture was centrifuged at 12,000× *g* for 10 min at 4 °C. The supernatant obtained from the centrifugation was collected for further analysis of T-AOC. Then, 3 mL of the FRAP reagent, which is prepared fresh on a daily basis, was combined with 100 μL of a diluted sample. After thorough mixing of the reagents, the A value at 593 nm was documented following a 30 min incubation period at a temperature of 37 °C.

The H_2_O_2_ content was determined as described by Sima et al. [47]. The frozen tissues of leaves and roots were homogenized in cold acetone. After homogenization, the resulting mixture was centrifuged at 5000 r min^−1^ for 5 min at 4 °C. The supernatant obtained from the centrifugation was collected for further analysis of H_2_O_2_. Then, 1 mL of the sample to be measured was absorbed and 5% titanous sulfate and concentrated ammonia water were added. After the formation of precipitation, the mixture was centrifuged at 5000 r min^−1^. Sulfuric acid (2 mol) was added to the precipitate. Colorimetric determination was performed at a wavelength of 410 nm.

### 4.4. Data Analysis

#### 4.4.1. Plant Selenium Transport Factor (TF) and Bioconcentration Factor (BCF)

The selenium TF and BCF were estimated among the soil and above-ground plant parts as [48]:

$$TF_{\mathrm{root-stem}}=Se_{\mathrm{stem}}/Se_{\mathrm{root}}$$

$$TF_{\mathrm{stem-leaf}}=Se_{\mathrm{leaf}}/Se_{\mathrm{stem}}$$

$$TF_{\mathrm{leaf-spike}\;\mathrm{glume}}=Se_{\mathrm{spike}\;\mathrm{glume}}/Se_{\mathrm{leaft}}$$

$$TF_{\mathrm{spike}\;\mathrm{glume-grain}}=Se_{\mathrm{grain}}/{Se}_{\mathrm{spike}\;\mathrm{glume}}$$

$$BCF_{\mathrm{root}}=Se_{\mathrm{Root}}/{Se}_{\mathrm{soil}}$$

$$BCF_{\mathrm{leaf}}=Se_{\mathrm{leaf}}/{Se}_{\mathrm{soil}}$$

$$BCF_{\mathrm{stem}}=Se_{\mathrm{stem}}/{Se}_{\mathrm{soil}}$$

$$BCF_{\mathrm{spike}\;\mathrm{glume}}=Se_{\mathrm{spike}\;\mathrm{glume}}/{Se}_{\mathrm{soil}}$$

$$BCF_{\mathrm{grain}}=Se_{\mathrm{grain}}/{Se}_{\mathrm{soil}}$$

where Se_root_, Se_stem_, Se_leaf_, Ses_pikeglume_, and Se_grain_ are the concentrations (μg kg^−1^, dry weight) of Se in the roots, stems, leaves, and grain, respectively, and Se_soil_ is the total selenium content in the 0–20 cm topsoil layer (μg kg^−1^).

#### 4.4.2. Soil Concentration Enrichment Factor (CEF)

The CEF method is a double normalized calculation method for quantitatively evaluating the effects of external factors on surface soil elements. By using the enrichment factor method, uncertainties due to factors such as sample collection, analysis, and distance from the pollution source can be eliminated, thereby making the results more reliable and accurate compared with the usual concentration comparisons. In this study, we utilized the CEF method to describe the characteristics of selenium enrichment in topsoil, where CEF was calculated according to Gong et al. [49] as

$$CEF=\frac{{\left(C_{i}/C_{j}\right)}_{Topsoil}}{{\left(C_{i}/C_{j}\right)}_{Subsoil}}$$

where C_i_ is the content of element i in a topsoil or subsoil sample and C_j_ is the content of fixed element j in a topsoil or subsoil sample.

The selection of fixed elements is a crucial step when calculating CEF because the fixed elements must be characterized as chemically stable. Elements that are susceptible to biological factors or that migrate and transform with changes in the physical and chemical conditions in the environmental medium are not suitable for use as fixed elements. The background values for soil elements in China (China National Environmental Monitoring Centre, Beijing, China) [50] and the background values for soil elements in Shanxi Province are presented in Table 3. Considering their stability, cobalt (Co), nickel (Ni), and lead (Pb) were selected as fixed elements. However, nickel (Ni) and lead (Pb) are readily influenced by external factors in the soil, such as industrial residues and agricultural fertilizers [51], and thus cobalt was selected as the fixed element for this study.

#### 4.4.3. Mobility Factor (MF)

The flow coefficient (MF) representing the relative flow index for selenium was used to assess the bioavailability of selenium in soil [52]. MF was calculated using the following formula, where F1 represents the ratio of water–Se relative to soil total selenium and F2 represents the ratio of exchangeable Se relative to total soil selenium.

$$MF=\left(F1+F2\right)/\sum_{i=1}^{5}Fi$$

#### 4.4.4. Statistical Analysis

Data were processed using Excel 2013 software (Microsoft Corp., Redmond, WA, USA) and figures were prepared with Origin 2021 (OriginLab, Hampton, MA, USA). The data were expressed as average values. SPSS 22.0 statistical analysis software (IBM Corp., Armonk, NY, USA) was used to perform the least significant difference test to compare cultivars at α = 0.05. Path analysis was performed using Amos 25 (IBM Corp., Armonk, NY, USA) structural equation modeling software. The experimental design diagram was drawn with Figdraw (https://www.figdraw.com/).

## 5. Conclusions

In this study, we analyzed the vertical distribution characteristics of soil selenium, the forms of soil selenium present, and the transport of selenium in black wheat in a selenium-rich area. We investigated the effects of nitrogen fertilizer application on these factors in the soil–black wheat system. We found available phosphorus, alkali-hydrolyzed nitrogen, and organic carbon contents were significantly positively correlated with the selenium contents under different nitrogen application rates. The pH value was negatively correlated with the soil selenium contents under high nitrogen application rates. We also found that the vertical distribution of soil selenium was influenced by two main pathways. The first pathway involved the influence of the soil layer on soil selenium through its effects on the total selenium content and subsequently on the forms of selenium (MF). The second pathway involved the influence of the soil layer on the alkali-hydrolyzed nitrogen content, which then affected the forms of selenium (MF). Further analysis of the plow layer and sub-tillage layer indicated that nitrogen application increased the proportions of water-soluble and exchangeable selenium among the total selenium, thereby making it more readily available for absorption by black wheat. Consequently, nitrogen application in a selenium-rich area affected the concentration and accumulation of selenium in black wheat grains by altering the forms of soil selenium as well as the wheat yield. We also conducted a black wheat cultivation test using different sodium selenite concentrations, which confirmed that nitrogen application enhanced the antioxidant capacity of wheat leaves, increased the activity of GSH-Px, alleviated selenium poisoning, and facilitated the transportation of selenium to the grain. By controlling the nitrogen fertilizer input in selenium-rich areas at an appropriate rate, we can effectively regulate the selenium level in the grains, thereby providing a scientific basis for the biofortification of wheat with selenium while maintaining high and stable yields.

## Figures and Tables

**Table 1:** Changes in environmental factors in selenium-rich soil from 0–100 cm layer under different nitrogen application rates.

N Application Rate	Soil Layer	Olsen-P	pH	Moisture	TOC	Alkaline-N	Total Se
(kg hm^−2^)	(cm)	(mg kg^−1^)		(%)	(g kg^−1^)	(mg kg^−1^)	(μg kg^−1^)
N0	0–20	56.82	7.53	10.11	18.18	67.67	723.06
	20–40	42.27	7.57	9.67	14.78	46.67	647.77
	40–60	18.28	7.58	10.03	9.31	51.33	810.15
	60–80	3.26	7.54	10.88	5.11	46.67	491.36
	80–100	1.61	7.44	11.02	2.53	30.33	201.56
N144	0–20	66.28	7.54	9.90	18.42	77.00	825.95
	20–40	34.12	7.56	8.78	15.93	72.33	754.04
	40–60	18.18	7.60	7.22	12.47	65.33	673.14
	60–80	2.99	7.52	7.94	11.22	53.67	642.60
	80–100	2.44	7.50	7.08	4.29	37.33	317.20
N168	0–20	67.23	7.57	9.26	10.53	81.67	734.12
	20–40	40.37	7.55	10.15	9.36	46.67	651.35
	40–60	13.37	7.57	10.25	6.05	35.00	284.10
	60–80	4.08	7.53	10.26	4.00	30.33	216.20
	80–100	2.86	7.55	9.62	3.19	32.67	159.62
N192	0–20	59.91	7.02	10.32	12.44	86.33	558.47
	20–40	28.00	7.12	10.07	9.94	65.33	341.56
	40–60	12.87	7.15	9.30	7.49	44.33	263.30
	60–80	2.50	7.25	10.82	4.48	32.67	256.23
	80–100	1.97	7.34	9.23	2.86	21.00	194.88
N216	0–20	60.06	7.27	10.75	13.39	93.33	386.62
	20–40	36.94	7.36	9.71	9.35	65.33	362.26
	40–60	15.71	7.44	9.76	6.96	39.67	313.55
	60–80	1.09	7.44	10.14	5.14	44.33	216.24
	80–100	2.36	7.46	9.51	5.39	53.67	320.36
N240	0–20	59.88	7.34	9.06	18.96	102.67	470.41
	20–40	44.05	7.38	9.81	13.42	84.00	418.48
	40–60	14.05	7.29	9.93	10.35	51.33	348.23
	60–80	2.03	7.44	11.08	8.34	28.00	347.45
	80–100	1.15	7.46	11.22	5.20	56.00	168.10

**Table 2:** Effects of nitrogen application in selenium-rich area on Se transport factor (TF) and bioenrichment factor (BCF) values in different wheat tissues.

Nitrogen Application Rate	TF_root-stem_	TF_stem-leaf_	TF_stem-spike glume_	TF_leaf-spike glume_	TF_spike glume-grain_	TF_stem-grain_	TF_leaf-grain_	BCF_root_	BCF_stem_	BCF_leaf_	BCF_spike glume_	BCF_grain_
N0	0.17	1.95	1.68	0.86	3.35	5.62	2.89	0.226	0.039	0.075	0.065	0.218
N144	0.19	1.88	2.14	1.14	3.22	6.91	3.68	0.139	0.027	0.050	0.057	0.185
N168	0.08	5.14	3.20	0.62	3.17	10.14	1.97	0.122	0.009	0.048	0.030	0.095
N192	0.46	1.42	1.37	0.96	3.68	5.02	3.54	0.128	0.058	0.082	0.079	0.292
N216	0.11	6.03	3.05	0.51	3.29	10.05	1.67	0.170	0.018	0.109	0.055	0.181
N240	0.05	15.04	8.40	0.56	1.95	16.38	1.09	0.122	0.006	0.097	0.054	0.105

**Table 3:** Background levels of soil element in Shanxi Province, China (mg kg^−1^).

Soil Layer	Co	Cr	Cu	Mn	Ni	Pb	V	Zn
Top_soil_	9.9	61.8	26.9	554	32	15.8	68.3	75.5
Sub_soil_	10.8	61.5	28.3	573	33.7	18.1	70.6	71.9

## Data Availability

The data that support the findings of this study are available from the corresponding author.
